# Supplementary figures and images for: GFP Reporter System Reveals Cell-to-Cell Variability in Aquaporin-2 Expression
Source: bioRxiv. 2025 Dec 18:2025.12.16.694664. Preprint. [Version 1] doi: 10.64898/2025.12.16.694664 (PMC12724717; doi:10.64898/2025.12.16.694664)

B

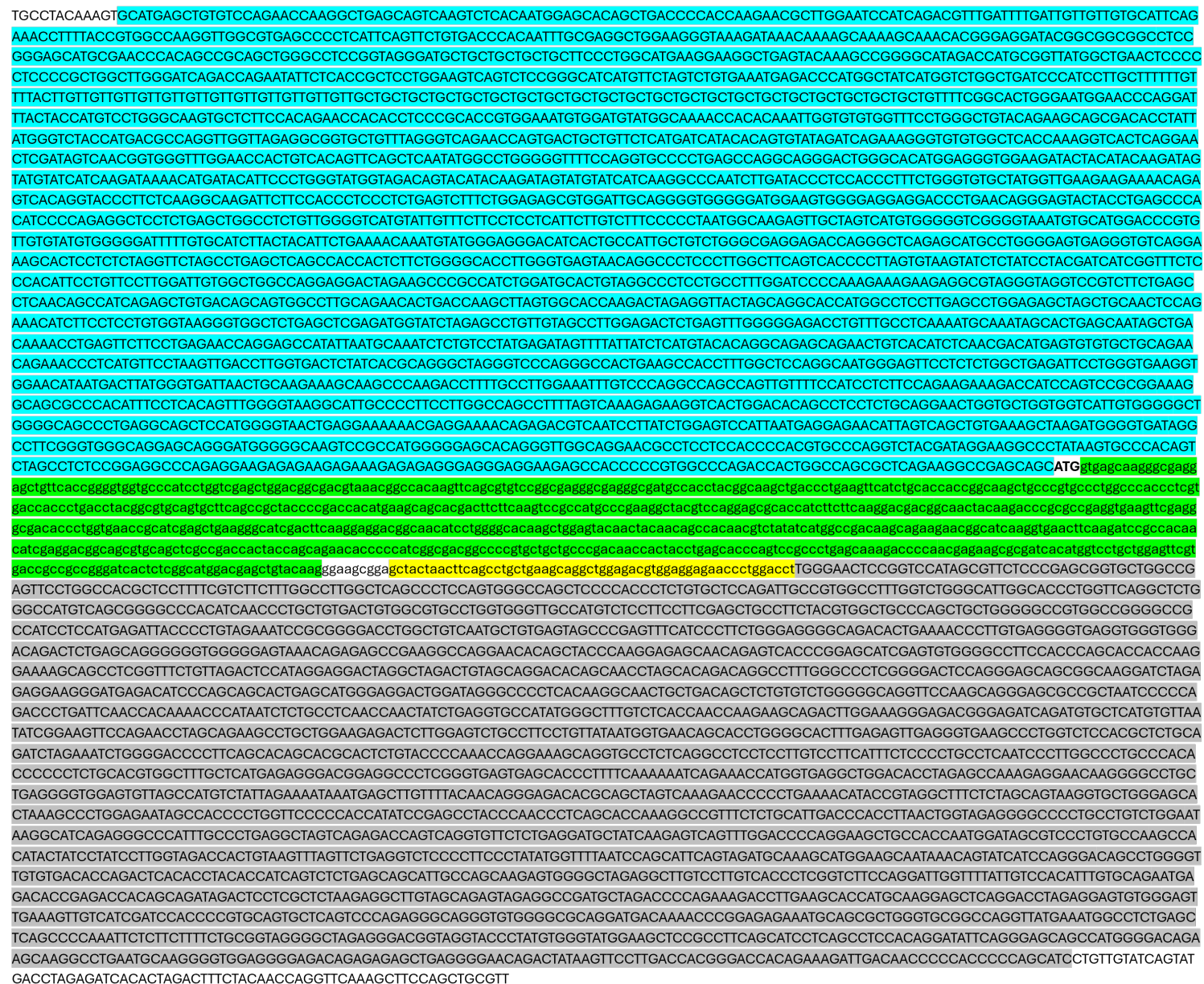

5' arm — GFP — P2A — 3' arm

Supplement: Supplement 1 [file media-1.pdf]

A

1 nM dDAVP

- + - + - + - + - + - + - + - + - + - +

50 kD →  
37 kD →

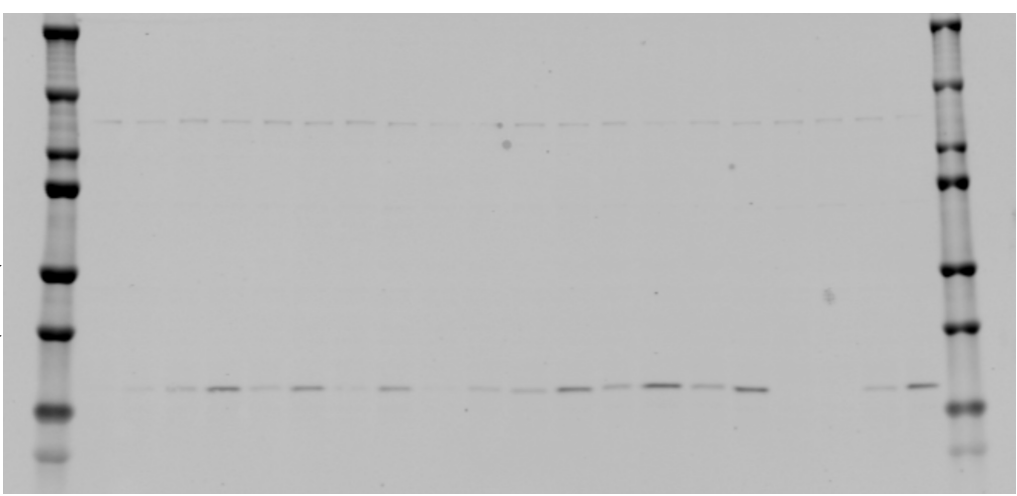

GFP

1 nM dDAVP

- + - + - + - + - + - + - + - + - + - +

50 kD →  
37 kD →

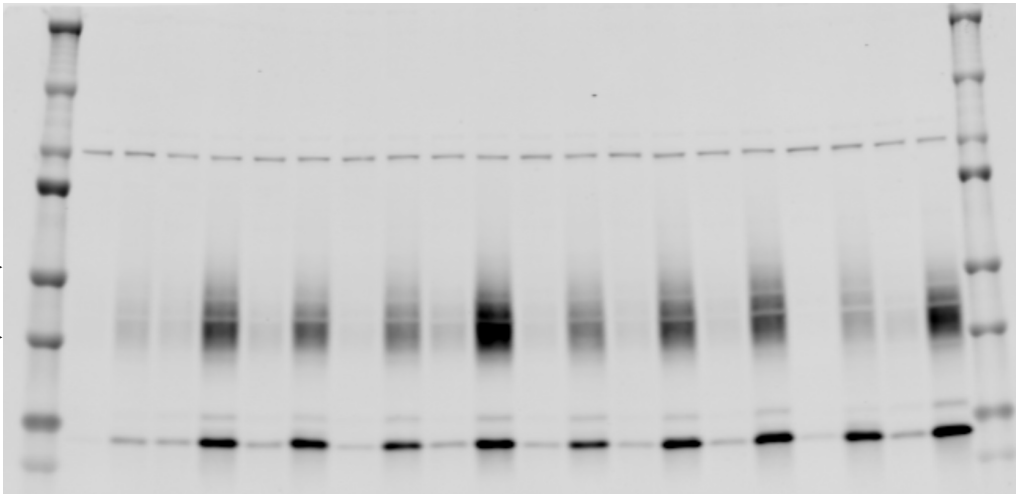

Aqp2

Supplement: Supplement 2 [file media-2.pdf]
